# Zebrafish Vascular Mural Cell Biology: Recent Advances, Development, and Functions

**DOI:** 10.3390/life11101041

**Published:** 2021-10-03

**Authors:** Koji Ando, Tomohiro Ishii, Shigetomo Fukuhara

**Affiliations:** Department of Molecular Pathophysiology, Institute of Advanced Medical Sciences, Nippon Medical School, Tokyo 113 8602, Japan; t-ishii@nms.ac.jp (T.I.); s-fukuhara@nms.ac.jp (S.F.)

**Keywords:** zebrafish, mural cells, pericytes, vascular smooth muscle cells, live imaging, development

## Abstract

Recruitment of mural cells to the vascular wall is essential for forming the vasculature as well as maintaining proper vascular functions. In recent years, zebrafish genetic tools for mural cell biology have improved substantially. Fluorescently labeled zebrafish mural cell reporter lines enable us to study, with higher spatiotemporal resolution than ever, the processes of mural cell development from their progenitors. Furthermore, recent phenotypic analysis of *platelet-derived growth factor beta* mutant zebrafish revealed well-conserved organotypic mural cell development and functions in vertebrates with the unique features of zebrafish. However, comprehensive reviews of zebrafish mural cells are lacking. Therefore, herein, we highlight recent advances in zebrafish mural cell tools. We also summarize the fundamental features of zebrafish mural cell development, especially at early stages, and functions.

## 1. Introduction

Blood vessels are mainly composed of vascular endothelial cells, that make up the luminal surface, and vascular mural cells, that cover the endothelial cells from the abluminal side. The recruitment of mural cells to the vascular wall plays an important role in angiogenesis, acting in an organ-dependent manner, as well as subsequent vascular stabilization, and functions. Mural cells are divided into two main types: pericytes and vascular smooth muscle cells (VSMCs) [1,2]. Pericytes mainly cover capillaries, whereas VSMCs cover relatively large vessels from arteries to small arterial vessels and veins. At the transition site between VSMCs and pericytes, pericytes are further categorized into several subtypes depending on their morphology or gene expression along the vascular tree [3]. However, recent single-cell RNA sequences of mouse brain mural cells revealed that pericytes form the single homogeneous population distinguished from VSMC clusters and indicated the lower genetic continuity between VSMCs and pericytes in the brain [2]. This single-cell RNA sequencing analysis also revealed several pericyte selective genes such as *Abcc9* and *Kcnj8* [2]; however, these genes expressions are present not only in pericytes, but also in VSMCs, and may even be absent in the pericytes of some organs [2,4]. A precise definition distinguishing these two cell types is needed, but thus far, clear and simple criteria for pericytes, which could be applied to all organs, have not been established [2,4]. Given the difficulty of establishing definitions and/or appropriate terminology, we use the term “mural cells” in this review, referring especially to those observed in early developmental stages, rather than using the terms “pericyte” or “VSMC” unless well-defined or used in the original reference sources.

The zebrafish is a suitable model animal for studying cardiovascular system development [5]. Zebrafish embryos are fertilized and develop externally, they are transparent, and can easily be manipulated genetically. Therefore, many transgenic zebrafish lines have been established for visualizing processes or intracellular signaling during vascular development [5,6,7,8,9,10]. These tools have dramatically expanded our knowledge of the processes underlying vasculogenesis and angiogenesis including sprouting, lumenization, and pruning. However, transgenic zebrafish reporters for visualizing all types of mural cells have not been available to investigators, hampering our understanding of the complex processes underlying mural cell development and subsequent vascular maturation steps. It is noteworthy, however, that several mural cell reporter lines have been developed in the last decade in our and other labs (Table 1), allowing us to achieve a better understanding of mural cell development and functions [11,12,13,14,15,16,17,18,19,20,21,22,23,24,25,26,27,28].

To the best of our knowledge, although there are many excellent reviews on other systems [29,30,31,32], there have been no comprehensive reviews focusing specifically on zebrafish mural cell development and functions thus far. Therefore, to facilitate optimal usage of the zebrafish as a model for mural cell biology, this review summarizes the fundamental processes of mural cell development, focusing especially on the early developmental stages, their regulatory mechanisms, and functions in zebrafish.

## 2. Genetic Tools for Mural Cell Biology in Zebrafish

**Figure 1:**
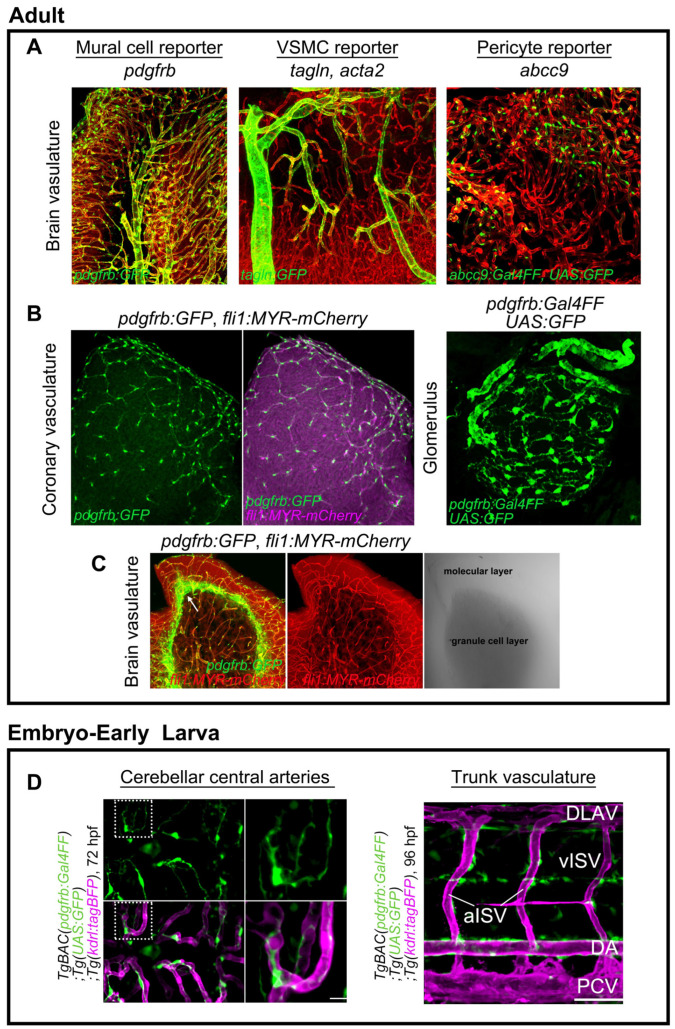
Transgenic zebrafish fluorescent reporter lines for mural cells. (**A**) Expressions of *TgBAC(pdgfrb:GFP)^ncv22Tg^* (left), *TgBAC(tagln:EGFP)^ncv25Tg^* (center), and *TgBAC(abcc9:GAL4FF)^ncv34Tg^* (right) reporters in zebrafish adult brain. Vessels are labeled with *Tg(fli1:MYR-mCherry)^ncv1Tg^* or *Tg(kdrl:DsRed2)^pd27^* (red). All types of mural cells are labeled with *pdgfrb.* VSMCs can be labeled with *tagln* or *acta2* reporter. Brain pericytes are selectively labeled with the *abcc9* reporter [2]. See also Table 1. (**B**) *pdgfrb* reporter labels mural cells in coronary vessels and glomerulus mesangial cells. (**C**) Non-vascular-associated *pdgfrb* expression (green) at the interface between the molecular and granule cell layers. Images are transverse sectional views of the adult zebrafish cerebellum. (**D**) *pdgfrb* reporter (green) labels mural cells in the brain and trunk from the early stages of development. Scale bars: 50 μm, 20 μm (enlarged images).

**Figure 2:**
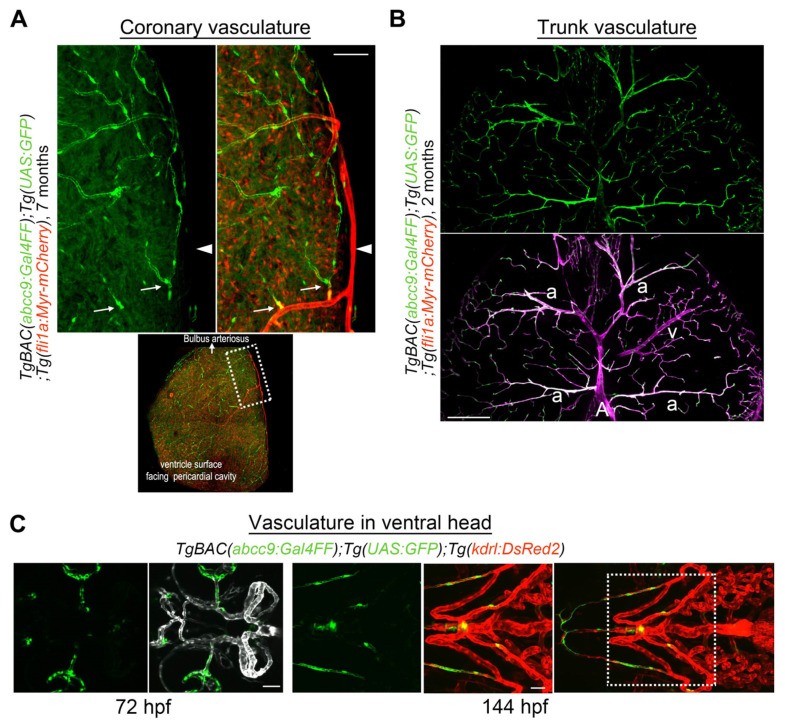
*abcc9* reporter expression in the zebrafish. (**A**) Expression of *TgBAC(abcc9:GAL4FF)^ncv34Tg^* (green) is selective in capillary pericytes in coronary vessels. *abcc9* reporter expression is negative around arteries (arrowhead) but starts after the appearance of several branches (arrows). Dotted area on the ventricle surface facing the pericardial cavity is enlarged at the top. (**B**) Transverse sectional view of the trunk vessels. In contrast to the brain (Figure 1A) and coronary vessels (Figure 2A), *abcc9* is positive in arteriolar VSMC (a), while still being negative in arterial VSMC covering the primary artery (A) or the dorsal aorta (not shown in this image). Interestingly, the expression of *abcc9* is lower in veins (v) than in arterioles or capillaries, which is also in clear contrast to the brain vasculature, suggesting that *abcc9* expression reflects organotypic differences among mural cells. (**C**) Observation of the *TgBAC(abcc9:GAL4FF)^ncv34Tg^* reporter (green) around the vasculature in the ventral head at 72 hpf (left) or 144 hpf (right) indicates that mural cells covering the ventral artery and aortic arches are negative for *abcc9*, which is similar to the findings in the dorsal aorta. Dotted area in the right image is enlarged on the left. Scale bars: 50 μm (A), 200 μm (B), 30 μm (C, 72 hpf), 50 μm (C, 144 hpf).

Zebrafish VSMC, a type of mural cells, were first described by Santoro et al. in a 2009 study utilizing an antibody for sm22a-b (*tagln*) and transmission electron microscopy [33]. In line with their observations, two transgenic zebrafish lines, *Tg(sm22a-b:GFP)* [18] and *Tg(acta2:GFP)* [23], were developed and shown to allow visualization GFP (green fluorescent protein)-positive mural cells suggestive of VSMCs in large-caliber vessels such as the dorsal or ventral aorta, but not in relatively small-caliber trunk vessels or cranial vessels, during embryonic stages. This prompted investigators to question the presence of pericytes in zebrafish [23]. Wang et al., on the other hand, succeeded in visualizing pericytes in brain vessels by performing fluorescence in situ hybridization for *platelet-derived growth factor receptor beta (pdgfrb)* [34]. In the trunk regions, *pdgfrb*-positive mural cells were detected not only in the ventral portion of the dorsal aorta, but also in the intersegmental vessels (ISVs). These results support the suitability of *pdgfrb* for visualizing all types of mural cells in zebrafish. Consistently, transgenic zebrafish carrying a modified BAC clone containing the *pdgfrb* promoter labeled all mural cells including pericytes and VSMCs in the organs, from the early to the adult stages (Figure 1A,B,D) [11,12]. It is noteworthy that mural cell precursors are weakly positive for *pdgfrb* and *pdgfrb* reporter lines, thereby enabling us to trace the specification process into the mural cell lineage from their *pdgfrb*^low^ precursors [12,28]. In addition to VSMC-selective and pan mural cell reporter lines, a pericyte-selective reporter for *abcc9*, which is a pericyte-selective gene found in the mouse brain, has been established (Figure 1A) [2,28]. We showed *abcc9* to be the earliest and most selective marker for mural cells, especially in the brain, and to become selective for pericytes during development (Figure 1A) [2,28]. In addition to the brain, *abcc9* reporter expression is found in coronary vascular pericytes (Figure 2A). In contrast, *abcc9* becomes positive in the trunk (arteriolar) VSMC (Figure 2B). These observations indicate organotypic differences among mural cells in zebrafish, as reported in mice [4]. These zebrafish mural cell reporters also indicate that fundamental gene expression profiles of mural cell types in the organs are conserved between zebrafish and mice [2]. Utilizing these novel genetic tools (Table 1), we can now easily address mural cell developmental processes from the early to the adult stages, including specification into mural cells, one of the major advantages of zebrafish as compared to other animal models.

Non-vascular-associated strong *pdgfrb* reporter expression is observable in the adult brain, including the stem cell niche area located at the cerebellum interface between the granule cell and molecular layers [35] (Figure 1D). PDGFRβ is reportedly expressed by neural stem cells in the adult mouse subventricular zone [36] as well as by human placental mesenchymal stem cells [37]. Thus, in addition to the established vascular biology, *pdgfrb* reporter zebrafish are also useful in a broad range of research fields.

## 3. Mural Cell Development in Zebrafish

### 3.1. Overview of Mural Cell Development during the Early Developmental Stages in Zebrafish

**Figure 3:**
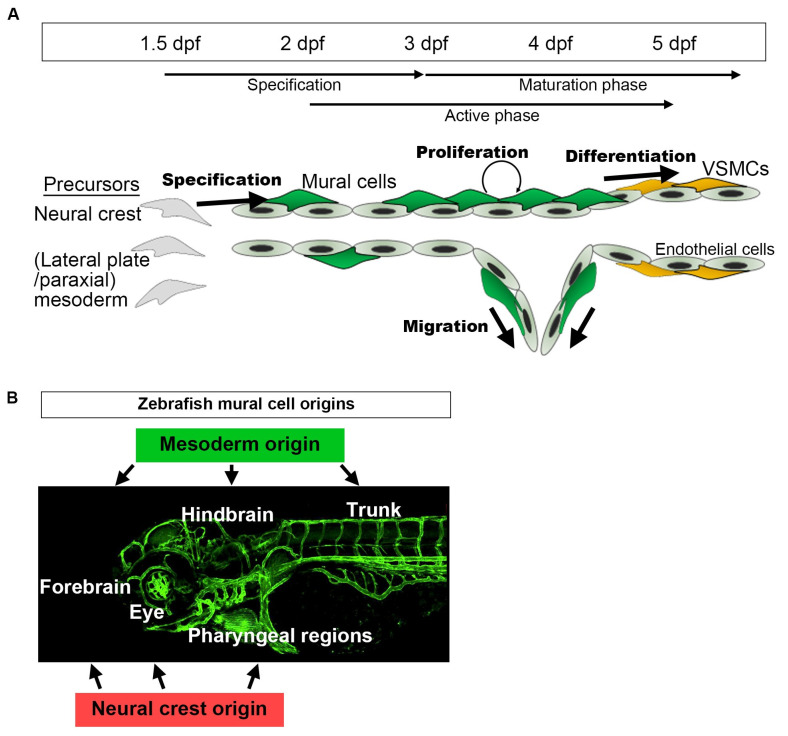
A schematic presentation of the developmental time course of zebrafish mural cells mainly in the brain and trunk. (**A**) Neural crest or mesoderm-derived mural cell precursors located in the vicinity of (arterial) endothelial cells are destined for the mural cell lineage during the period 36–72 hpf. Subsequently, such mural cells actively proliferate and migrate to cover arterial vessels. This proliferation and migration of mural cells appears to take place when active angiogenesis is induced (active phase). Along with the establishment of a vascular hierarchy, mural cells on larger caliber vessels start to differentiate into VSMCs after approximately 72 hpf (maturation phase). Upon formation of the vascular system, mural cell proliferation, migration, and differentiation take place. Mural cells, other than those on the dorsal/ventral aorta, resemble pericytes when they first appear, especially in the brain. However, whether they are identical to pericytes or still undergoing maturation (progenitor) to differentiate into VSMCs or pericytes remains unknown. (**B**) Mural cells in the hindbrain and trunk are of mesoderm origin, and those in pharyngeal regions and eyes are from the neural crest. The anterior part of the brain (forebrain) contains mural cells derived from both the mesoderm and the neural crest [12].

We will first summarize the basic developmental time course of zebrafish mural cells, mainly in the trunk and brain vasculature, and then move on to individual tissues and organs. *pdgfrb*-positive cells are initially observed at approximately 10 h post-fertilization (hpf), and these *pdgfrb*-positive mural cell precursors are broadly distributed in the trunk or cerebral base by 24–48 hpf [12]. Among them, those located in proximity to arteries are specified to the mural cell lineage during the period spanning approximately 36–72 hpf [28] (Figure 3A). Increased *pdgfrb* expression or the induction of *abcc9* is the hallmark of specification into the mural cell lineage [28]. However, most of the mural cells beneath the dorsal aorta and approximately half of those in ISVs are negative for *abcc9* by 5 days post-fertilization (dpf) [28], suggesting differences in mural cell characteristics from the first step of development onward. After specification, mural cells actively proliferate and migrate toward the arteries not covered with mural cells, and almost all of the brain and trunk arteries have been covered by mural cells by 4–5 dpf. Simultaneously, mural cells around large-caliber vessels such as those comprising the Circle of Willis and the dorsal aorta start to express *tagln* or *acta2* and appear to acquire VSMC properties after 3 dpf. At this early age, VSMC markers were reportedly detectable in only a subset of the mural cells in ISV and were barely detected in those of central arteries (CtAs) before 5 dpf [28]. The mural cells lacking VSMC markers show pericyte features morphologically (protruded round cell body and extension of long thin processes longitudinally along vessels) and genetically (positive for the pericyte marker *abcc9*). However, whether these cells are actually pericytes or still undergoing maturation needs to be confirmed. During the early developmental stages, although the primordial hindbrain channel (PHBC) has few, most of veins lack mural cell coverage. As yet, little is known about how and when zebrafish veins acquire mural cell coverage. Lineage tracing employing the Cre/lox-system and genetic intervention leading to the loss of specific cell derivatives revealed mural cells in the trunk, forebrain, and hindbrain to be derived from the mesoderm, whereas those in the forebrain, hyaloid vessel, and pharyngeal regions are from the neural crest (Figure 3B) [12].

### 3.2. Mural Cell Development in Axial Vessels in the Trunk

**Figure 4:**
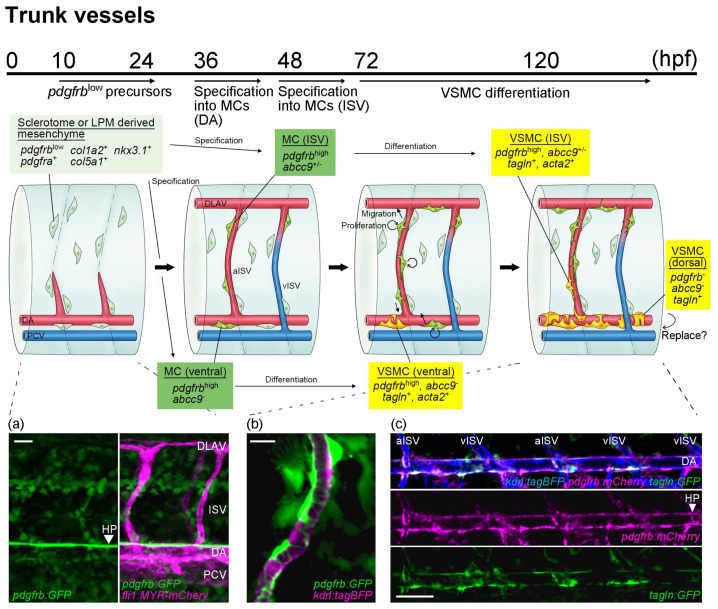
A schematic presentation of mural cell development in trunk vessels during early developmental stages. Potential *pdgfrb*^low^ mural cell precursors (light green) are detectable from 10 hpf and are distributed throughout the trunk by 24 hpf, preferentially along the somite boundary where ISV is formed (**a**). Then, specification from *pdgfrb*^low^ precursors located in the vicinity of arterial endothelial cells into mural cells (green) starts after approximately 36 hpf beneath the dorsal aorta and 48 hpf on the ISVs. Subsequently, the proliferation and migration of mural cells are both induced in a PDGFRβ signaling-dependent manner. The arterial ISV (aISV) (**b**), the dorsal aorta (**c**), and the dorsal portion of the venous ISV (vISV) are well covered by mural cells by 96 hpf. Mural cells beneath the dorsal aorta start to wrap around the dorsal aorta and express VSMC markers such as *acta2* and *tagln* at approximately 72 hpf. Later, these VSMC markers become positive in some ISV mural cells. In most mural cells differentiating into VSMC, *acta2* expression is induced earlier than that of *tagln.* Images obtained in the vicinity of the dorsal aorta of *TgBAC(pdgfrb:mCherry) ^ncv23Tg^;TgBAC(tagln:GFP)^ncv25Tg^;Tg(kdrl:tagBFP)^mu293Tg^* at 96 hpf are shown (**b**). Interestingly, the expression of *tagln*;GFP in mural cells increases when these cells are combined with *pdgfrb* reporter lines. Mural cells that are *tagln*-positive but negative for *pdgfrb* become apparent on the dorsal side of the dorsal aorta, mostly at approximately 96 hpf. However, whether VSMCs on the dorsal aorta consist of both VSMC sources or are replaced by mural cells emerging from the dorsal side, as observed in the mouse, remains to be determined. Scale bars: 20 μm (**a**,**b**), 50 μm (**c**).

Trunk mural cells are derived from sclerotomes and the lateral plate mesoderm. Time-lapse imaging performed by Rajan et al. clearly showed that *nkx3.1*-positive sclerotomes and sclerotome-derived notochord-associated cells disperse around the trunk region, and that some are distributed along the ISVs by 48 hpf [38]. These sclerotome-derived mesenchymal cells in the vicinity of arterial endothelial cells in ISVs subsequently become mural cells (Figure 4). In addition to a low level of *pdgfrb* expression, these sclerotome-derived cells are positive for *pdgfra* and extracellular matrix genes, *col1a2* and *col5a1*, suggesting these cells to have fibroblast characteristics [38]. Considering the onset of mural cell emergence in ISV after 48 hpf [12,28], the specification into mural cells from sclerotome-derived fibroblasts takes place after the alignment of progenitors around arterial vessels. Given how well distributed those precursors are around ISVs [38], there might be signals actively recruiting *pdgfrb*^low^ mesenchymal precursors to ISV walls (or somite boundaries) rather than stochastic phenomena. Among perivascular *pdgfrb*^low^ cells, only a few become mural cells. Therefore, mural cells increase their number and their vascular coverage via subsequent proliferation and migration. After the important specification period (36–72 hpf) in the trunk, the perivascular *pdgfrb*^low^ mesenchyme not specified to become mural cells shows decreased *pdgfrb* expression [28]. Whether there is communication between mural cells and perivascular fibroblasts during their development remains unknown.

The mural cells beneath the dorsal aorta can be seen after approximately 36 hpf, earlier than ISV mural cells. Subsequently, these dorsal aorta mural cells start the process of entirely covering the dorsal aorta and after 3 dpf they express VSMC markers, *acta2* [23] and/or *tagln* [20], indicating differentiation into VSMC (Figure 4). Although half of ISV mural cells are negative for *abcc9* before 5 dpf, nearly all mural cells beneath the dorsal aorta are consistently negative for *abcc9* when they first become detectable [28]. Thus, dorsal aorta mural cells appear to be programed to be VSMC from the beginning of the specification process. In addition to the ventral side, *tagln*-positive cells are observed on the dorsal side of the dorsal aorta [20], becoming prominent a few days after the emergence of mural cells at the ventral side. Different from those emerging on the ventral side, most *tagln*-positive cells on the dorsal side are negative for *pdgfrb* reporter expression [12]. Therefore, distinct types of VSMCs might be present on the dorsal and ventral sides of the dorsal aorta. Consistent with this possibility, the lateral plate mesoderm-derived VSMCs in mice reportedly cover the ventral side of the descending dorsal aorta in the early stage of aortic development, whereas the VSMCs covering the dorsal side are from paraxial mesoderm-derived somites [39]. Eventually, all dorsal aorta VSMCs are replaced by somite-derived VSMCs [39]. The emergence of ISV mural cells was strongly suppressed when the production of paraxial mesoderm derivatives was inhibited by *tbx6* morpholino oligonucleotide (unpublished data), whereas that of mural cells beneath the dorsal aorta was suppressed when paraxial and lateral plate mesoderm derivative productions were both inhibited [12]. Therefore, ISV mural cells are mainly derived from paraxial mesoderm and dorsal aorta mural cells are from both paraxial and lateral plate mesoderm, observations consistent with the results of previous studies [20,38]. To date, the reasons for VSMC on the dorsal aorta arising from different sources and whether there are differences in their roles remain unknown. As reviewed by Sato [40], endothelial cells in the dorsal aorta show dorsoventral differences in their origins. Using quail-chick chimera analysis, Pardanaud et al. demonstrated that the dorsal portion of the dorsal aorta is composed of somite-derived endothelial cells and splanchnic mesoderm-derived endothelial cells at the ventral portion of the dorsal aorta [41]. This study was further extended, and it was shown that the hemogenic ventral wall of the dorsal aorta is replaced by non-hemogenic somite-derived endothelium in chicks [42]. In zebrafish, the dorsoventral difference within the dorsal aorta is well documented, especially during hemogenic endothelial cell development [43,44]. Therefore, dorsoventral differences in the dorsal aorta may affect mural cell emergence and the subsequent characteristics of these cells, in addition to explaining the difference in origins. However, there is no evidence of a dorsoventral endothelial cell lineage difference in zebrafish as reported in chicks. Rather, this is likely to reflect local differences in signaling [45,46,47], as shown in mice [48]. Interestingly, *pdgfrb* signaling reportedly promotes hematopoiesis in the ventral part of the dorsal aorta in zebrafish [49,50]. Therefore, the dorsoventral differences in VSMC might contribute to the efficient progress of hematopoiesis. Future analysis of ventral and dorsal VSMC gene profiles is anticipated to provide better understanding of their physiological roles and the well-programed events of vascular development.

### 3.3. Mural Cell Development in the Brain

**Figure 5:**
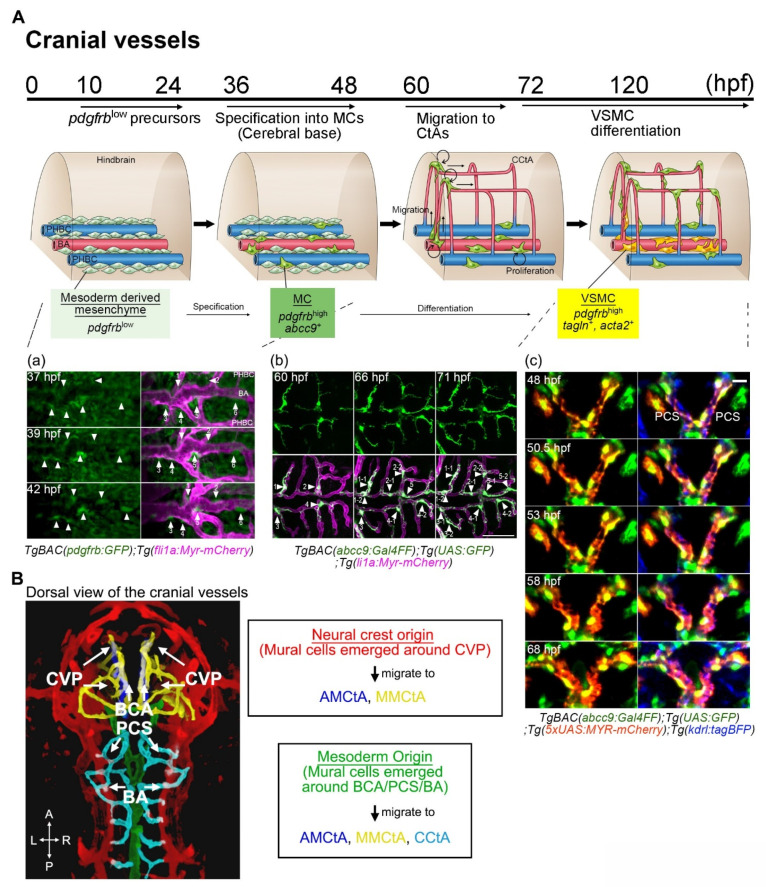
(**A**) A schematic of mural cell development in cranial vessels during early development from 10 hpf and are distributed at the cerebral base and around the CVP by 24 hpf. Then, specification from *pdgfrb*^low^ precursors located in the vicinity of arterial endothelial cells on the BA, PCS, BCA, or CVP into mural cells (green) starts at approximately 36 hpf (**a**). Specification into mural cells from precursors is also slightly induced around primordial hindbrain channels (PHBCs). After the connection of CtAs to CVP, BCA, PCS, or BA and the start of blood circulation, mural cells emerging at the cerebral base migrate to CtAs. When these cells migrate, cell division frequently take place to entirely cover CtAs (**b**). Time-lapse imaging of *TgBAC(abcc9:Gal4FF)^ncv34Tg^;Tg(UAS:GFP);Tg(5xUAS:MYR-mCherry)^ncv504Tg^;Tg(kdrl:tagBFP)^mu293Tg^* shows morphological changes from pericyte-like cells to VSMCs around the PCS (**c**). Mural cell bodies and plasma membranes are highlighted by GFP (green) and MYR-mCherry (magenta), respectively. This VSMC-like morphological change in the cerebral base precedes the expression of VSMC markers such as *acta2* or *tagln*. This is not always the case in CtA mural cells, however. (**B**) Mural cells appearing around the CVP are derived from the neural crest, whereas those emerging around the BCA, PCS, and BA have a mesodermal origin. Reflecting the connection routes of vessels and the features of mural cell migration along the vessels, mural cells around the CVP migrate toward AMCtAs (blue) and MMCtAs (yellow); those appearing at the BCA to AMCtAs and MMCtAs; and those emerging around the PCS or BA toward CCtAs (aqua). Therefore, mural cells in the zebrafish forebrain, in the hindbrain, or in the middle tend to become neural crest, mesoderm, or mixed-origin tissues, respectively. Scale bar: 20 μm (**c**). Choroidal vascular plexus (CVP). Basal communicating artery (BCA). Posterior communicating segment (PCS). Basilar artery (BA). Primordial hindbrain channel (PHBC).

Embryonic brain mural cells are derived from the *pdgfrb*^low^ mesenchyme distributed in the cerebral base at the level of the basilar artery (BA), just above the anterior endodermal cell sheet, or around the choroidal vascular plexus (CVP) [12] (Figure 5A). Similarly to axial vessels, the *pdgfrb*^low^ mesenchyme located in the vicinity of endothelial cells is specified to the mural cell lineage after approximately 36 hpf (Figure 5A). These mural cells were observed to emerge at the cerebral base or CVP, then proliferate, and after CtAs had connected to these vessels and blood flow had begun, migrate to CtAs along the vessels. When they migrate, these cells preferentially extend their processes along inter-endothelial junctions [12]. Interestingly, they form scaffold-like structures in the extending processes at the inter-endothelial cell junctions and 90% of mural cells were observed to move forward by relocating their cell bodies to this node, as if they had jumped from the original point to this node [12]. When the cells migrate to the next position, mural cell division frequently takes place simultaneously and leaves one mural cell at the original position while supplying the additional mural cell to the uncovered new region, thereby expanding vascular coverage. However, whether this is also the case when a mural cell co-migrates with endothelial tip cells during sprouting angiogenesis is unknown. Once angiogenesis in the brain has stabilized at around 5 dpf, mural cell migration and proliferation are also arrested [12], indicating the behaviors of endothelial and mural cells to be synchronized. This correlation between mural cells and endothelial proliferation is reportedly observed during wound healing angiogenesis in the skin of adult zebrafish [51].

The mural cells emerging around the CVP migrate toward anterior mesencephalic central arteries (AMCtAs) and the middle mesencephalic central artery (MMCtA) along the connecting vessels between the CVP and AMCtA/MMCtA (Figure 5B). Mural cells, in contrast, emerging on the basal communicating artery (BCA), primarily migrate to the AMCtA and MMCtA, the posterior communicating segment (PCS) to the MMCtA, and the BA to CCtAs (Figure 5B). Mural cells emerging around the CVP are derived from the neural crest, whereas those around the Circle of Willis and BA are from the mesoderm; therefore, zebrafish hindbrain mural cells are mainly of mesoderm origin in both the forebrain and the intermediate region (Figure 5B) [12]. These distinct brain region populations persist in the adult brain [12]. Thus, the comparison of gene expressions between mural cells derived from the neural crest and those from the mesoderm in the zebrafish brain provides clues to elucidating the mechanisms regulating the organotypicity of mural cells (“organ environment” vs. “origin”).

In the embryonic brain, *abcc9* is the earliest and most selective marker for specified mural cells. Soon after the specification into mural cells, *abcc9* reporter expression is induced in nearly all of the mural cells in the brain [28] and shows typical pericyte-like morphology (Figure 5A). Subsequently, mural cells at the Circle of Willis and BA transform morphologically and show a shape typical of VSMC (Figure 5A). In accordance with the morphological changes, these mural cells start to express *tagln* and *acta2* after approximately 72 hpf [28,31], suggesting differentiation into VSMCs. Before reaching the Circle of Willis and BA, mural cell expressions of *acta2* and *tagln* are induced around the caudal division of the internal carotid (CaDI) [52], whereas those of mural cells around CtAs begin later. VSMC marker expression in AMCtA- or CCtA-covering mural cells starts with the cells located close to the branching point from the BCA or BA, respectively, after 5–6 dpf [53]. Thus, VSMC marker expression gradually turns toward the downstream vessels (CaDI → Circle of Willis → CtAs) during development, which may indicate that mechanical forces produced by blood flow facilitate differentiation into VSMCs, given the importance of blood flow in VSMC development in zebrafish, as described below [19,24]. Blood flow was not essential, however, for specification into mural cells and the *de novo* emergence in zebrafish trunk and brain vessels [12]. Brain mural cells, especially around the CtAs, initially show typical pericyte morphology and a gene signature characterized by *abcc9*, but later differentiate into VSMCs around large-caliber vessels, which is different from the dorsal aorta mural cells. Contractile VSMCs reportedly have low migratory and proliferation ability [54]. Considering the active migration and proliferation of mural cells during embryonic stages to entirely cover brain vessels, this mural cell development in the brain passing through a pericyte-like cell type stage before the differentiating into VSMC appears to be both reasonable and beneficial. This system, probably not only in the brain but also in other organs and tissues, may allow the spatio-temporal flexibility of VSMC coverage in accordance with vascular development. It would be interesting and potentially worthwhile to investigate the gene expressions related to cell motility and the proliferation of both cell types. As yet, we do not know whether these pericyte-like mural cells observed during early embryonic stages are identical to what we consider to be (mature) pericytes or immature progenitors of pericyte and VSMCs. Detailed gene expression analysis during the course of development may answer this question.

### 3.4. Mural Cell Development in the Ventral Head

Mural cells are observed around the ventral aorta from approximately 66 dpf, and these cells arise mainly from a *foxc1b*+ neural crest derivative [23,24]. Mural cells in the ventral aorta and aortic arches become positive for *acta2* but negative for *abcc9* soon after their initial appearance (Figure 2C), suggesting that the mode of differentiation is similar to that of VSMC observed in the dorsal aorta. Interestingly, most ventral aorta mural cells are also negative for *pdgfrb* [24], as observed in the dorsal part of the dorsal aorta.

### 3.5. Mural Cell Development in the Kidney

Glomerular mesangial cells, known to be a type of mural cell, are clearly visualized in *pdgfrb* reporter zebrafish (Figure 1A). Glomerular development in the zebrafish pronephros starts after 34 hpf and vascularization in the glomerulus becomes visible after 2 dpf [55]. However, the precise timing of mesangial cell emergence and the mechanisms underlying zebrafish mesangial development, including its origin, remain unknown [55].

### 3.6. Mural Cell Development during Regeneration

Observation of adult zebrafish skin tissue over a few months demonstrated that neither endothelial cells nor pericytes divided or migrated [51]. This suggests that endothelial cells and pericytes are in a quiescent state in normal skin capillaries, thereby maintaining a stable vascular structure. However, endothelial cells were rapidly activated by wounding, and angiogenesis was induced in non-injured blood vessels. During angiogenesis in skin wound healing, pericytes also increased in number by proliferating at a rate similar to that of endothelial cells and migrated to the leading edge [51]. According to the termination of neovascularization (active angiogenesis process), the number of endothelial cells and pericytes started to decrease gradually and returned to the pre-injury level over the course of a few months, indicating the synchronized regulation of endothelial cell and pericyte dynamics. The source of mural cells in the regenerated vessels was analyzed in a fin regeneration model [15]. Tracking photo-converted cells unexpectedly revealed that pre-existing mural cells are not the source of those for repaired vessels [15]. In the zebrafish fin, mural cells originate from *pdgfrb*-positive cuboidal-shaped cells around arterial vessels running through the center of the fin ray, in regenerating vessels but also in normal development. Furthermore, Pdgfrb signaling turned out to be essential for the *de novo* emergence of mural cells from cuboidal-shaped cells during the process of regeneration. However, whether mural cells in the regenerating vessels are always supplied by *de novo* emergence during the repair process at the other sites is unknown.

## 4. Molecular Mechanisms Underlying Mural Cell Development

### 4.1. PDGFRβ-Mediated Signaling in Mural Cell Development

**Figure 6:**
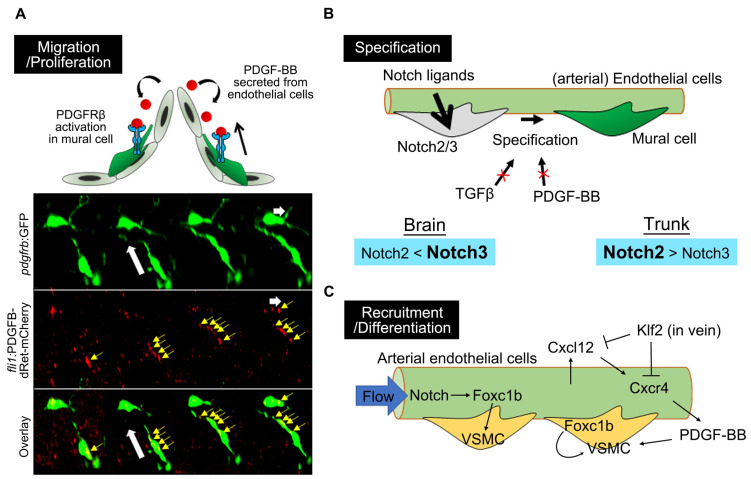
Molecular mechanisms underlying zebrafish mural cell development. (**A**) PDGF-BB/PDGFRβ signaling is essential for zebrafish mural cell migration and proliferation. The timelapse imaging of brain mural cells during the embryonic stage, at the bottom, shows the uptake of mCherry-fused PDGF-B into the mural cell processes (yellow arrows) extending toward the direction of migration. White arrows indicate the direction of mural cell migration. mCherry-fused PDGF-B depleted of the retention motif was originally expressed by endothelial cells using the *fli1* promoter. This observation fits the proposed model in which PDGF-BB secreted from endothelial cells activates PDGFRβ expressed in mural cells to attract these cells to the vascular wall and the leading front. (**B**) Notch2/3 are both indispensable for mural cell specification, with a preference for Notch3 in the brain and Notch2 in the trunk. TGFβ or PDGF-BB is not essential for specification into mural cells, at least during early developmental stages, in the brain or trunk vessels of zebrafish. (**C**) Arterial endothelial cells induce VSMC recruitment or VSMC differentiation via Foxc1b or in a PDGF-BB-dependent manner. Cell-autonomous Foxc1b function, in VSMC developing from progenitors, has also been reported.

Our recent study utilizing *pdgfb* and *pdgfrb* mutant zebrafish revealed that the organotypic requirement of PDGFB–PDGFRβ signaling for mural cell development recognized in studies of mice is similar to that in zebrafish, suggesting a highly conserved role for PDGFB–PDGFRβ signaling in mural cell recruitment in vertebrate species [53]. As noted above, mural cells expand their vascular coverage via migration and proliferation. It is known that PDGF-BB released from endothelial cells activates PDGFRβ expressed on mural cells, thereby promoting mural cell migration/recruitment to the vascular wall and their proliferation [56]. *pdgfb* or *pdgfrb* mutant zebrafish analysis confirmed that PDGFB–PDGFRβ signaling is not essential for mural cell specification, although is indispensable for subsequent migration and proliferation [53,56,57]. *In vivo* live imaging in zebrafish showed that the uptake of PDGF-B released from endothelial cells into the mural cell processes extended toward the direction of migration (Figure 6A), which further supports the proposed model that PDGF-B functions as a chemoattractant for mural cells via PDGFRβ [56]. In zebrafish, the Pdgfb ligand-encoding *pdgfb* gene is duplicated, i.e., to *pdgfba* and *pdgfbb*, both of which are enriched in endothelial cells. *pdgfba*;*pdgfbb* double-mutants showed additive mural cell loss, although with a greater contribution from *pdgfba* than *pdgfbb*, suggesting that *pdgfba* and *pdgfbb* function cooperatively [53]. The higher contribution of *pdgfba* than *pdgfbb* probably reflects their expression levels in endothelial cells [53]. A recent study also showed that Cxcr4 activation in arterial endothelial cells induces Pdgfb production, whereas this pathway is blocked by Klf2 in venous endothelial cells, thereby recruiting mural cells to the dorsal aorta, but not to veins [58]. However, it is not clear whether the *de novo* formation of mural cells around the dorsal aorta is affected or differentiation into VSMC from the mural cell lineage is inhibited because analyses were performed using *tagln* and *acta2* VSMC reporters, in which the first *pdgfrb*-positive mural cells to emerge were not labeled.

*pdgfb* and *pdgfrb* mutant analyses indicated this signaling to be indispensable for the *de novo* formation of mural cells via specification from the perivascular mesenchyme in the trunk and brain vessels [12,53]. However, there is a discrepancy between the *pdgfrb* mutant phenotype and previous zebrafish studies regarding trunk mural cells and vascular development, as discussed in detail in a recent publication [53]. Briefly, *pdgfrb* mutants display normal vascular development throughout the body and VSMC coverage beneath the dorsal aorta during the early stages of development. However, inhibition of Pdgfrb signaling by the ubiquitous over-expression of a dominant negative form of Pdgfrb [20], morpholino [59], or an inhibitor [59] resulted in defective ISV formation [59] or decreased VSMC coverage of the dorsal aorta [20]. Considering the possibility that dominant negative proteins can interfere with related molecules and that non-sense-mediated decay of the *pdgfrb* transcript may upregulate compensatory paralogous genes [60], this discrepancy raises the possibility of the existence of redundant or compensatory pathways for mural cell development [60,61]. One candidate for such a function is the related PDGFRα receptor, encoded by the *pdgfra* gene. However, *pdgfra*;*pdgfrb* double-mutant larvae at 4 dpf showed neither trunk vascular defects nor any significant reduction in the VSMC coverage of the dorsal aorta as compared to the wild type [53], although some *pdgfra* mutant larvae without flow showed VSMC loss, apparently reflecting an essential role in VSMC development [19]. These results suggest that *pdgfra* and *pdgfrb* are dispensable for initial development of the trunk vasculature and VSMCs on the dorsal aorta at this stage. Therefore, the discrepancy might not be explained by the compensatory role of Pdgfra, acting as a substitute for Pdgfrb, in mural cell development; further studies are needed to elucidate the mechanisms responsible for the observed differences.

### 4.2. Notch Signaling in Mural Cell Development

The molecular mechanisms underlying mural cell development, particularly the specification step, have yet to be clarified. In efforts to understand the molecular mechanisms regulating mural cell specification, zebrafish serve as a useful model because in vivo processes can easily be traced from precursors. It was found that mural cell emergence was fully inhibited by treatment during the specification period (36–72 hpf) with γ-secretase inhibitors, i.e., DAPT and LY411575, that block Notch signaling [28]. Furthermore, mural cell emergence is completely prevented in embryos depleted of both *notch2* and *notch3*, which were found to be expressed in mural cells [28]. Suppression of either *notch2* or *notch3* expression did not completely inhibit mural cells, suggesting that these two genes function redundantly during specification into mural cells (Figure 6B). Timelapse imaging of zebrafish Notch activity reporters clearly demonstrated Notch activation in mural cells during specification [28]. *pdgfrb*^low^ mesenchymal cells in which Notch activity is “ON” show increased *pdgfrb* expression, which is one of the hallmarks of specification, and extend their processes along the endothelial cells to assure the formation of close contacts, which would be a typical morphological feature of mural cells. However, the cells without Notch activation do not show such changes and *pdgfrb* expression is further reduced. These results indicate that *pdgfrb*^low^ mesenchymal cells are specified to mural cells through the activation of Notch2 and Notch3 signaling, probably via their ligands expressed in arterial endothelial cells, as shown by a mouse study [62,63]. Therefore, lower expression of Notch ligands in venous endothelial cells [62,63,64] might explain the absence of mural cell coverage in veins during early developmental stages. Zebrafish mural cells arise from the mesoderm and neural crest [12], but Notch2 and Notch3 signaling are essential for mural cell specification regardless of their origin [28]. However, Notch3 functions predominantly in brain mural cells and Notch2 in trunk mural cells, suggesting that the properties of mural cells differ depending on the site of action starting in the very early stage of development. Chen et al. demonstrated blood-flow-induced Notch activation in arterial endothelial, i.e., not only in mural cells, to be important for the recruitment of *acta2*- or *tagln*-positive VSMC to the dorsal aorta [19]. However, it is not clear whether this Notch activation regulates the *de novo* formation of mural cells or subsequent differentiation into VSMC. A prior study found that Notch3 morphants showed cranial hemorrhage, considered to be attributable to a reduction in brain mural cells [34]. However, neither cranial hemorrhage nor edema is induced, even in the absence of mural cells in the CtAs, as reported in *pdgfrb* mutants [12,53]. This suggests that cranial hemorrhage in embryos lacking Notch3 might be induced by defects in endothelial cells because Notch3 also functions in these cells [64].

### 4.3. Forkhead Box Transcription Factors

Forkhead box domain transcription factors such as Foxc1 [65] and FoxF2 [66] are expressed by pericytes in the mouse brain and regulate pericyte proliferation and blood–brain barrier (BBB) formation. These genes are also important for zebrafish mural cell development [19,24,25,67]. Duplicated *foxc* genes in zebrafish, *foxc1a and foxc1b*, and genetic deletion or suppression by morpholinos of these genes results in impaired VSMC coverage of the ventral artery [24]. *foxc1b* is expressed in neural-crest-derived progenitors and positively regulates subsequent differentiation into VSMCs in the ventral portion of the vessel [24]. *foxc1a* is important for *foxc1b* expression, such that the *foxc1a* mutant shows a stronger effect than the *foxc1b* mutant on VSMC differentiation around the ventral artery [24]. On the other hand, Chen et al. revealed that *foxc1b* induced by flow-mediated Notch activation in arterial endothelial cells promotes VSMC recruitment, whereas *foxc1a* suppression in endothelial cells exerted no major effects on VSMC coverage in the dorsal aorta [19]. Hence, *foxc1* functions in both mural cells and endothelial cells to promote VSMC development (Figure 6C). The zebrafish *foxf2* gene is also duplicated, as *foxf2a* and *foxf2b*, and both genes are expressed in brain mural cells as well as endothelial cells [67]. Interestingly, *foxf2b* mutants show reduced *pdgfrb*-positive pericytes and *acta2*-positive VSMC in the zebrafish brain [67]. A murine study revealed that FoxF2 is important for PDGFRβ expression [66]. There are thus several possible mechanisms by which *foxf2b* regulates mural cell development in zebrafish, i.e., migration and proliferation driven by Pdgfrb, specification to the mural cell lineage, and even subsequent differentiation processes, whether cell-autonomously or non-cell-autonomously, via endothelial modulation.

### 4.4. TGFβ Signaling

Tumor growth factor β (TGFβ) signaling is also among the well-known factors involved in mural cell differentiation, survival, and proliferation, both via the TGFβ and activin branches of the pathway [68,69,70,71]. However, TGFβ signaling turned out to largely be dispensable for zebrafish brain and trunk mural cell development during the early developmental stages, at least through 6 dpf [28]. Neither pharmacological inhibition of TGFβ signaling nor the depletion of *alk1* was associated with major problems in mural cell specification. Additionally, a transgenic zebrafish reporter that monitors the activation of TGFβ or activin/nodal signaling revealed activation of this pathway in endothelial cells, but there was no induction in either brain or trunk mural cells in the early stages (unpublished data). Although reductions in mural cell number were observed on brain vessels and ISVs following treatment with SB431542 or *alk1* morpholino, this might represent indirect effects. Similarly, *alk5* mutants exhibit defective VSMC development in the cardiac outflow tract, but this is considered to be attributable to secondary effects of perturbed endothelial TGFβ signaling [72].

## 5. Function of Mural Cells in Zebrafish

### 5.1. Observations Made in the Phenotyping Portion of pdgfrb Mutant Zebrafish Studies

**Figure 7:**
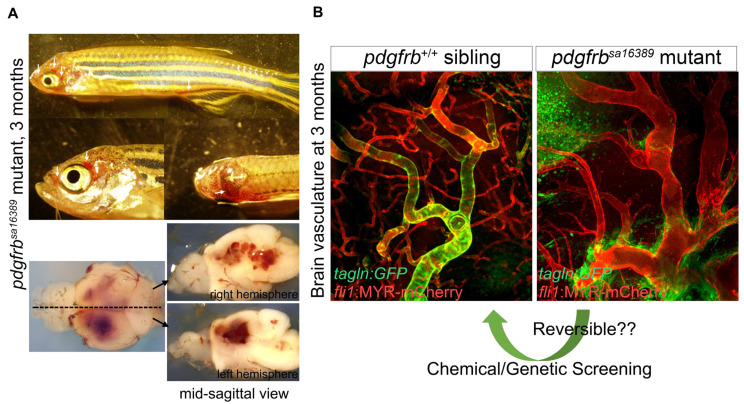
Abnormal vascular formation in *pdgfrb* mutant. (**A**) *pdgfrb^sa16389^* mutants have no apparent vascular abnormalities, edema, or signs of hemorrhage during early developmental stages [12,53]. They start to show clear vascular abnormalities, however, after 1 month of age. After the juvenile stage, they have signs of bleeding (arrows) and the associated prominent phenotype in brain vessels. Images of brains dissected from the fish shown on the top are presented in the lower panels. (**B**) Comparison of the brain vasculature of wild-type (left) and *pdgfrb^sa16389^* mutant (right) mice, showing the same anatomical region, confirms the capillary network reduction and dilation of arteries. Coverage with VSMC visualized by *TgBAC(tagln:EGFP)^ncv25Tg^* reporter (green) found in the wild type is absent in the *pdgfrb* mutant. Regardless of how severe vascular defects are, *pdgfrb* mutant zebrafish can reach adulthood and produce viable offspring, which is a unique feature of zebrafish and indicates the utility of the *pdgfrb* mutant zebrafish for chemical or genetic screening, allowing the discovery of treatments for vascular anomalies such as aneurysms. Scale bars: 50 μm (B). Fluorescent images are from [53].

Phenotyping studies of *Pdgfb* and *Pdgfrb* mutant mice lacking pericyte coverage have made major contributions to elucidating the functions of pericytes. Recently, we carried out extensive phenotypic analyses of *pdgfrb* mutant zebrafish organs from the early embryonic through adult stages (see details in Ando et al., 2021) [53]. Although *pdgfrb* mutant zebrafish do not have developmental defects during the early stages, they later exhibit severe vascular pathology (Figure 7). We found that the organ-specific differences in the sensitivity of mural cell recruitment to loss of *pdgfrb* in zebrafish correspond to those previously reported for Pdgfb and Pdgfrb mutant mice, suggesting a high degree of evolutionary conservation of these processes in vertebrates. In marked contrast to mice, however, zebrafish *pdgfrb* null mutants reach adulthood despite extensive cerebral vascular anomalies and hemorrhage (Figure 7A), offering unique opportunities to model cerebrovascular pathology and test therapeutic strategies. Taking advantage of the tractability of zebrafish for chemical/genetic screening and the resistance of this model to early death in the absence of pericytes, *pdgfrb* mutant zebrafish may prove useful in discovering and testing drugs for treating cerebrovascular as well as neurovascular diseases (Figure 7B).

### 5.2. BBB

Brain vessels acquire a specialized barrier property, the aforementioned BBB, to limit the passage of substances between blood and the parenchyma. The BBB is established by an endothelial cell sheet and surrounds other cell types, such as pericytes and astrocytes. Several independent studies have shown that zebrafish develop a BBB [73,74,75,76,77]. Imaging of reporter expression for *glut1* (earliest BBB marker) or *plvap* (which correlates negatively with BBB maturation) indicates that BBB formation starts when cerebral angiogenesis begins at 2 dpf [76]. Evaluation of BBB integrity by tracer leakage from the circulation into the brain parenchyma has further demonstrated that the BBB becomes sufficiently mature by 5 dpf [73], consistent with the timing of the formation of the fundamental cerebral vascular structure and the completion of mural cell coverage. Cerebral vascular leakage through transcytosis is high during early stages and is suppressed by 5 dpf, reflecting the induction of *mfsd2aa* expression in endothelial cells [73]. Consistent with the results of a mouse study, *mfsd2aa* mutant zebrafish persistently showed elevated brain vasculature permeability through adulthood [73], indicating the conserved role of Mfsd2a in the suppression of transcytosis, which is important for BBB integrity. It remains unknown, however, as to whether zebrafish pericytes positively control this Mfsd2a expression, as suggested by the mouse studies. In contrast to morpholino-based analysis [78], *mfsd2aa* mutant zebrafish did not exhibit a hemorrhagic phenotype in the brain [73] such as that observed in the Mfsd2a mutant mouse [79,80]. Mutants or morphants of *notch3* [34], *foxc1* [25], or *pitx2* [25] reportedly exhibit hemorrhage in the brain, which presumably results from defective mural cell development. However, to our surprise, loss of mural cell recruitment to CtAs in *pdgfrb* mutant zebrafish did not lead to vascular malformations or any apparent signs of hemorrhage during early developmental stages [53], indicating that endothelial cells can produce basic barrier integrity without pericytes in zebrafish. Therefore, the aforementioned hemorrhagic phenotype in mutant embryos may not be due to impaired mural cell recruitment, although whether the BBB is fully mature at embryonic stages without pericytes is an issue which merits more detailed investigation. *pdgfrb* mutant zebrafish began developing severe vascular malformations and hemorrhage in the brain at around one month of age. Why these defects arise so much later in zebrafish, despite similar mural cell loss at an early age, is unknown. As discussed in our recent report [53], differences in blood pressure or oxygen supply may explain these unexpected findings. At a later age, the vascular defects in zebrafish may progress showing courses similar to those in mouse embryos.

**Figure 8:**
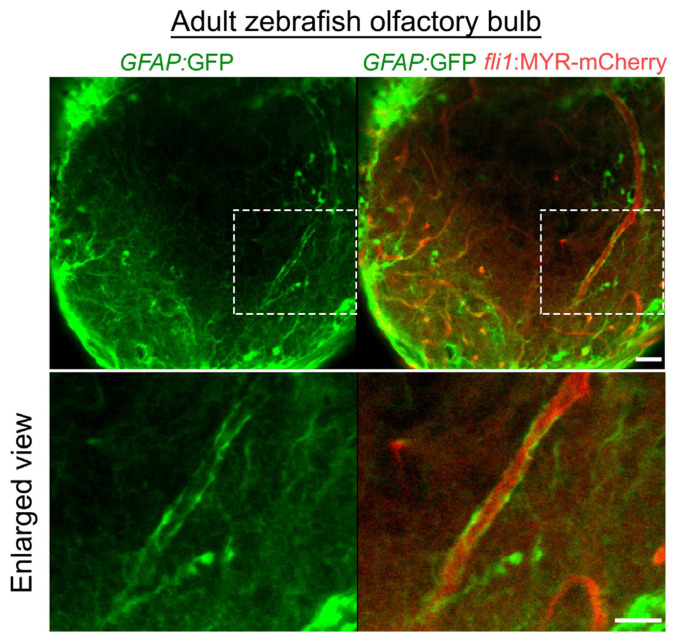
Distribution of astroglia in the zebrafish olfactory bulb. *GFAP*:GFP reporter (green) indicates vascular coverage by astroglia in the adult zebrafish olfactory bulb. Dotted areas are enlarged at the bottom. Scale bars: 30 μm, 20 μm (enlarged images).

Astroglial cells also contribute to BBB integrity. Astrocyte endfeet wrap around the vascular wall within the central nervous system, and endothelial cells develop into the BBB cooperatively with astrocytes. Whether zebrafish possess astrocytes similar to those of mammals has been questioned, because instead, zebrafish develop a type of glial cells with radial morphology often termed radial glia, which are labeled by glial fibrillary acidic protein (GFAP) [74,81,82]. However, a recent analysis revealed that these GFAP-positive cells have gene signatures and functions similar to those of mammalian astrocytes [81]. Furthermore, despite lacking the classical stellate astrocyte morphology seen in mammals, the endfeet of GFAP-positive cells envelop the vascular wall in the adult olfactory bulb (Figure 8), possibly indicating that zebrafish develop neurovascular units as in mammals [82]. However, extension of GFAP-positive cell endfeet to the vessels in other brain regions was not prominent in the early stages, including 5 dpf, when the BBB matures into the adult structure (unpublished data) [74]. This might be attributable to the unsuitability of GFAP for labelling astroglial cells associated with endothelial cells. Therefore, future analyses are required to elucidate the role of astroglial cells in zebrafish BBB formation.

### 5.3. Regulation of Vascular Tone

Zebrafish blood circulation starts on the dorsal aorta at ~24 hpf. After circulation begins, mural cells emerge beneath the dorsal aorta independently of blood flow and then differentiate into VSMCs in a blood-flow-dependent manner. Recruitment of mural cells is important for basement membrane organization around the dorsal aorta [20]. In the absence of mural cell coverage, basement membrane components such as collagen Ⅳ and fibronectin around the dorsal aorta are drastically reduced, which leads to dilation of the dorsal aorta and increased elasticity of the vascular wall [20]. In this setting, mRNA expression levels of collagen Ⅳ and fibronectin in endothelial cells are similar but fragmentation of these proteins is increased. Therefore, mural cells are important for basement membrane stabilization in addition to producing vascular membrane proteins. Acquisition and alteration of the vasoactivity of cerebral pericytes and VSMC during the early stages of development have been investigated [52]. At 4 dpf, soon after their emergence, VSMCs can induce vascular constriction in response to vasoconstrictors, whereas the vasodilation capacity of VSMC is not yet fully developed. Eventually, VSMCs become vasoactive, capable of both constriction and relaxation, by 6 dpf. On the other hand, capillary pericytes have the ability to regulate the vascular diameter in response to vasoconstrictors and vasodilators at 4 dpf, but lose this capacity by 6 dpf. The timing of VSMC coverage of trunk vessels, heralding the start of vascular diameter regulation via their constriction and relaxation, remains uncertain, but may progress as observed in the brain.

## 6. Closing Remarks

The importance of pericytes and VSMC in vascular formation and functions is well recognized, although the precise mechanisms governing the development of these cells under in vivo conditions remain as yet incompletely understood. In the 2010s, there were major advancements in our fundamental understanding of mural cell biology in zebrafish. A series of recent analyses revealed that the basic molecular mechanisms regulating mural cell development, as represented by PDGFRβ signaling, and the roles of mural cells are well conserved between zebrafish and mammals. Thus, the zebrafish is a powerful model organism for addressing mural cell development, dynamics, and functions. Combining mural cell reporters with techniques such as fluorescent biosensors, the Cre/lox-mediated lineage tracing system, and omics analysis, further understanding of the mechanisms governing mural cells develop will be achieved in the future. Additionally, mural cell reporters are useful for investigating cell-to-cell communication with endothelial cells, fibroblasts, and possibly other as yet unknown interacting cell types. Considering the relationships of developmental abnormalities affecting mural cells with cerebral disease, future extensive analyses utilizing zebrafish as a model to identify candidate genes functions for mural cell development will also yield better understanding of the processes of pathogenesis and provide insights for devising new clinical treatments.

## Figures and Tables

**Table 1:** Transgenic zebrafish lines for mural cells.

Regulatory Regions/Promoter	Types of Labeled Mural Cells	Expressed Gene	Name/Construct	Original Reference	ZFIN ID	Note
***pdgfrb***	**All mural cells (Pericyte, VSMC)**	mCitrin	*TgBAC(pdgfrb:Citrine)^s1010^*	Vanhollebeke et al., 2015 [11]	ZDB-TGCONSTRCT-150911-2	BAC clone; CH73-289D6. Inserted position of Citrin is different from that of GFP in *TgBAC(pdgfrb:EGFP)^ncv22Tg^*
		GFP	*TgBAC(pdgfrb:EGFP)^ncv22Tg^*	Ando et al., 2016 [12]	ZDB-TGCONSTRCT-160609-1	BAC clone; CH1073-606I16
		GFP	*TgBAC(pdgfrb:EGFP)^uq15bhTg^*	Bower et al., 2017 [13]	ZDB-ALT-180306-11	BAC clone; CH1073-606I16
		GFP	*Tg(pdgfrb:EGFP)^ue302Tg^*	Rider et al., 2017 [14]	ZDB-TGCONSTRCT-170830-1	Use 7.16-kbp *pdgfrb* promoter region
		mCherry	*TgBAC(pdgfrb:mCherry)^ncv23Tg^*	Ando et al., 2016 [12]	ZDB-ALT-160609-2	BAC clone; CH1073-606I16
		H2B-dendra	*Tg(pdgfrb:H2B-dendra)^mu158^*	Leonard et al., 2021 [15]	Not assigned yet	BAC clone; CH1073-606I16
		TetA-2A-AmCyan	*Tg(pdgfrb:TETA-2A-AmCyan)^mps7Tg^*	Tsata et al., 2021 [16]	ZDB-ALT-200915-4	CRISPR-Cas9-mediated targeted knock-in of CreERT2 at *pdgfrb* coding locus
		Gal4FF	*TgBAC(pdgfrb:GAL4FF)^ncv24Tg^*	Ando et al., 2016 [12]	ZDB-ALT-160609-3	BAC clone; CH1073-606I16
		Gal4-VP16	*Ki(pdgfrβ:Gal4)*	Xu et al., 2017 [17]	ZDB-ALT-171120-1	CRISPR/Cas9-mediated targeted knock-in of Gal4-VP16 at *pdgfrb* coding locus
		CreERT2	*Tg(pdgfrb:CreERT2)^mps6Tg^*	Tsata et al., 2021 [16]	ZDB-ALT-200911-6	CRISPR-Cas9-mediated targeted knock-in of CreERT2 at *pdgfrb* coding locus
***tagln***	**VSMC**	GFP	*Tg(tagln:GFP)^p151^*	Seiler et al., 2010 [18]	ZDB-ALT-101123-2	Use *tagln* promoter region containing ECR5
		GFP	*TgBAC(tagln:EGFP)^ncv25Tg^*	Ando et al., 2016 [12]	ZDB-ALT-160609-4	BAC clone; CH1073-307D13
		Caax-EGFP	*Tg(tagln:CAAX-EGFP)^uto37Tg^*	Chen et al., 2017 [19]	ZDB-ALT-170323-2	Use 2 kbp *tagln* promoter region
		NLS-EGFP-2A-CFP-FTASE	*Tg(tagln:NLS-EGFP-2A-CFP-FTASE)^y450Tg^*	Stratman et al., 2017 [20]	ZDB-TGCONSTRCT-170227-2	NA
		mCherry	*Tg(tagln:mCherry)^sh441Tg^*	Elworthy et al., 2019 [21]	ZDB-TGCONSTRCT-191111-1	Use *tagln* promoter region containing ECR5
		NLS-mCherry	*Tg(tagln:NLS-mCherry)^sh480Tg^*	Chhabria et al., 2019 [22]	ZDB-TGCONSTRCT-201222-1	NA
		ECR-GAL4	*Tg(tagln:ECR-GAL4)^y449Tg^*	Stratman et al., 2017 [20]	ZDB-TGCONSTRCT-170227-1	NA
***acta2***	**VSMC**	GFP	*Tg(acta2:GFP)^ca7Tg^*	Whitesell et al., 2014 [23]	ZDB-TGCONSTRCT-120508-1	Use 2.4-kbp *acta2* promoter region
		GFP	*TgBAC(acta2:EGFP)^uq17bh^*	Bower et al., 2017 [13]	ZDB-TGCONSTRCT-180306-9	BAC clone; DKEY-256C3
		mCherry	*Tg(acta2:mCherry)^uto5Tg^*	Chen et al., 2017 [19]	ZDB-TGCONSTRCT-170323-1	Use *acta2* promoter region
		mCherry	*Tg(acta2:mCherry)^ca8Tg^*	Whitesell et al., 2014 [23]	ZDB-TGCONSTRCT-120508-2	Use 2.4 kbp *acta2* promoter region
		Gal4FF	*Tg(acta2:GAL4FF,myl7:EGFP)^ca62Tg^*	Whitesell et al., 2019 [24]	ZDB-TGCONSTRCT-200102-2	Use 2.4 kbp *acta2* promoter region
***foxc1b***	**VSMC**	GFP	*Tg(foxc1b:EGFP)^mw44Tg^*	French et al., 2014 [25]	ZDB-TGCONSTRCT-150312-6	Use 5 kbp *foxc1b* promoter region
		EOS	*Tg(foxc1b:Eos)^tsu2013Tg^*	Qiu et al., 2016 [26]	ZDB-TGCONSTRCT-161212-5	Use 3.6 kbp *foxc1b* promoter region
		Gal4-VP16	*Tg(-5foxc1b:GAL4-VP16)^mw72Tg^*	Miesfeld et al., 2015 [27]	ZDB-TGCONSTRCT-151218-7	NA
		Gal4FF	*Tg(foxc1b:GAL4FF,myl7:EGFP)*	Whitesell et al., 2019 [24]	ZDB-TGCONSTRCT-200102-3	Use 5 kbp *foxc1b* promoter region
***myh11a***	**VSMC**	YFP	*Tg(myh11a:YFP)mu125*	Leonard et al., 2021 [15]	Not assigned yet	BAC clone; CH73-223E22
***abcc9***	**Pericyte, (VSMC in the trunk)**	Gal4FF	*TgBAC(abcc9:GAL4FF)^ncv34Tg^*	Ando et al., 2019 [28]	ZDB-TGCONSTRCT-210412-1	BAC clone; CH211-58C15. IRES-Gal4FF fragment is inserted at the c-tail of *abcc9* (ENSDART00000079987), which resembles SUR2B isoform. Construction method is described in Vanlandewijck et al., 2018 [2]. *abcc9* reporter become selective for pericyte in brain or coronary vessels, but arteriolar VSMCs are also labeled in the trunk.

## Data Availability

Not applicable.
